# Immunoexpression of TGF-β/Smad and VEGF-A proteins in serum and BAL fluid of sarcoidosis patients

**DOI:** 10.1186/s12865-015-0123-y

**Published:** 2015-10-06

**Authors:** Wojciech J. Piotrowski, Justyna Kiszałkiewicz, Paweł Górski, Adam Antczak, Witold Górski, Dorota Pastuszak-Lewandoska, Monika Migdalska-Sęk, Daria Domańska-Senderowska, Ewa Nawrot, Karolina H. Czarnecka, Zofia Kurmanowska, Ewa Brzeziańska-Lasota

**Affiliations:** Department of Pneumonology and Allergy, 1st Chair of Internal Medicine, Medical University of Lodz, Lodz, Poland; Department of Molecular Bases of Medicine, 1st Chair of Internal Medicine, Medical University of Lodz, 251 Pomorska St., 92-213 Lodz, Poland; Department of General and Oncological Pulmonology, 1st Chair of Internal Medicine, Medical University of Lodz, Lodz, Poland

**Keywords:** Sarcoidosis, TGF-β_1_, VEGF-A, Prognosis, Growth factors, Angiogenesis

## Abstract

**Background:**

The chronic course of pulmonary sarcoidosis can lead to lung dysfunction due to fibrosis, in which the signalling pathways TGF-β/Smad and VEGF-A may play a key role.

**Methods:**

We evaluated immunoexpression of TGF-β_1_, Smad2, 3, and 7, and VEGF-A in serum and bronchoalveolar lavage (BAL) fluid of patients (*n* = 57) classified according to the presence of lung parenchymal involvement (radiological stage I *vs.* II-III), acute *vs.* insidious onset, lung function test (LFT) results, calcium metabolism parameters, percentage of BAL lymphocytes (BAL-L%), BAL CD4^+^/CD8^+^ ratio, age, and gender. Immunoexpression analysis of proteins was performed by ELISA.

**Results:**

The immunoexpression of all studied proteins were higher in serum than in BAL fluid of patients (*p* >0.05). The serum levels of TGF-β_1_ (*p* = 0.03), Smad2 (*p* = 0.01), and VEGF-A (*p* = 0.0002) were significantly higher in sarcoidosis patients compared to healthy controls. There were no differences within the sarcoidosis group between patients with vs. without parenchymal involvement, acute vs. insidious onset, or patients with normal vs. abnormal spirometry results. In patients with abnormal spirometry results a negative correlation was found between forced vital capacity (FVC) % predicted value and TGF-β_1_ immunoexpression in BAL fluid, and positive correlations were observed between the intensity of lung parenchymal changes estimated by high-resolution computed tomography (HRCT scores) and Smad 2 level in serum.

**Conclusions:**

TGF-β/Smad signalling pathway and VEGF-A participate in the pathogenesis of sarcoidosis. BAL TGF-β_1_, and Smad 2 in serum seem to be promising biomarkers with negative prognostic value, but further studies are required to confirmed our observations.

## Background

Sarcoidosis is a chronic inflammatory disorder of unknown aetiology. The diagnosis is made based on a clinical and radiological picture, and is usually confirmed by the presence of non-caseating granulomas in involved organs. In about 90 % of patients granulomas are present in intrathoracic lymph nodes and/or lung parenchyma, but extrapulmonary presentations are frequent [1, 2]. The prognosis is relatively good; in about 60 % of patients with a plethora of phenotypes the disease disappears without any clinically significant remains. However, in other patients the course may be chronic, sometimes progressive, or recurrent. The most severe complication is lung fibrosis, occurring in 10–15 % of patients and leading to severe functional impairment, disability, and sometimes to death. Among different negative prognostic factors—lung interstitial disease, lung function test abnormality (of both restrictive and obstructive patterns), and severe impairment of calcium homeostasis may be listed as examples, whereas acute disease onset and isolated intrathoracic lymph node enlargement (radiological stage I) are considered good prognostic markers [3]. A tremendous research effort has been made to find a reliable biomarker that would be useful to predict long-term prognosis in sarcoidosis patients. Unfortunately, the results have been inconclusive, and it may be difficult at the disease onset to anticipate which patients would be free of disease and which would develop the progressive and fibrotic form in future. The role of TGFβ and TGFβ signalling pathway elements (SMADs) have been extensively studied in animal models of lung fibrosis and in idiopathic lung fibrosis (IPF), and this particular pathway seems to be critical in wound healing, scarring, and fibrosis in different organs and different diseases [4–6]. VEGF is a major contributor to angiogenesis and regulates several cell functions via its receptors (VEGFRs). The angiostatic-angiogenic axis (HIF-1a—VEGF—ING-4) may play a role in the pathogenesis of experimental lung fibrosis and IPF [7, 8]. Moreover, it was shown recently that these two molecular pathways are closely interrelated. For instance, in cultured human umbilical vein endothelial cells (HUVEC) physiological concentrations of VEGF attenuated TGF-β-related phosphorylation of Smad2/3 [9]. TGF-β1 has been shown to stimulate VEGF-A expression in human lung fibroblast via the Smad3 signalling pathway, but it downregulates VEGF-D expression through TGF-β receptor and JNK signalling pathway [10]. Interestingly, the same authors found decreased expression of VEGF-D in lung tissue of IPF patients [10]. In a rat model of lung fibrosis treatment with adenoviral delivery of VEGF resulted in reduced endothelial apoptosis, increased vascularisation, and decreased pulmonary hypertension due to reduced remodelling, but significantly worsened pulmonary fibrosis [7]. Therefore, the net effect of VEGF on lung fibrosis may depend on the isoform predominance, as well as the extent to which it is embedded in a cytokine network.

In sarcoidosis data are scarce and even more ambiguous. TGF-β_1_ concentration was increased in BAL fluid of sarcoidosis patients, but only in those with impaired lung function [11]. Contrary to this, polymorphic alleles of TGF-β_1_, implicated in lower levels of protein production, were associated with more severe disease presentation [12]. Other genetic studies indicate the role of polymorphic variants of TGF-β_3_ (with presumed modulating role on TGF-β_1_ activity) in sarcoidosis-related fibrotic lung disease [13], and a protective role of TGF-β_2_ SNP [14].

Smad proteins have not been studied in sarcoidosis so far. Data on the role of VEGF in the pathogenesis of sarcoidosis are also inconclusive. VEGF BAL concentrations were shown to be higher in IPF patients in comparison to sarcoidosis in one study [15], but higher than in IPF and hypersensitivity pneumonitis in another [16]. A study on induced sputum showed lower VEGF levels in sarcoidosis compared to healthy controls, and lower sarcoidosis in stage III-IV compared to stage I [17]. Contrary to this, in another study both serum and BALF VEGF levels were increased in sarcoidosis in comparison to controls [18]. This variability of results between different studies may be related to the fact that VEGF is involved in angiogenesis and lymphangiogenesis in the early stages of sarcoid inflammation and is not directly involved in fibrosis, so the results may be different depending on the stage of the process, the phenotype (acute vs. chronic), and the different representation of such processes as formation of granuloma and scarring in the same patient.

In view of the above, the following questions are intriguing to us: 1. Are the immunoexpressions of these proteins different for sarcoidosis and healthy subjects when measured in serum and BAL fluid 2?. What are the relations between these proteins in serum and BAL fluid? 3. Are there any differences within the sarcoidosis group between patients with different phenotypes, and in relation to selected prognostic markers?

## Methods

### Study and control groups

A total of 57 patients with pulmonary sarcoidosis were recruited into the study. Patients were admitted to the Department of Pneumonology and Allergy of Norbert Barlicki Memorial University Hospital No. 1 in Lodz during the years 2010–2014. The diagnosis was made based on current guidelines [1]. For each patient a consistent clinical and radiological picture of sarcoidosis with the presence of non-caseating granuloma in tissue biopsy was confirmed. The diagnosis was documented by EBUS-TBNA, bronchial mucosal biopsy, transbronchial peripheral lung biopsy, videomediastinoscopy, or extrathoracic biopsy (skin, peripheral lymph nodes). The biopsy was not obligatory in patients with bilateral hilar lymph node enlargement, acute symptoms (Löfgren syndrome), and typical BAL results (increased percentage of lymphocytes with CD + 4/CD + 8 > 3.5). In the whole sarcoidosis group the diagnosis was confirmed by biopsy in 42 patients. Based on chest X-ray results, patients were classified into the following radiological subgroups: stage I (hilar lymph node enlargement without signs of parenchymal involvement), stage II (signs of parenchymal involvement in addition to hilar lymph node enlargement), stage III (parenchymal involvement without visible hilar lymph node enlargement), and stage IV (signs of irreversible extensive lung fibrosis). Independent comparison of patients with acute disease onset (Löfgren syndrome with arthritis, erythema nodosum, elevated body temperature—with at least two symptoms present) and patients with insidious disease onset was made. The clinical and biological characteristics of the study group are presented in Table 1.

**Table 1:** Clinical and biological characteristics of the study group. Patients were grouped according to the absence/presence of lung parenchymal changes on chest X-ray (stage I vs. stage II-III) and clinical phenotype (acute vs. insidious onset)

	Stage I	Stages II-III	Acute onset	Insidious onset
	*n* = 30	*n* = 27	*n* = 29	*n* = 28
Gender	15 F 15 M	11 F 16 M	15 F 14 M	11 F 17 M
Age	38.63 ± 8.84	42.88 ± 11.64	37.29 ± 12.06	36.62 ± 7.12
FEV_1_ [% pred.]	94.75 ± 14.01	92.30 ± 15.33	96.81 ± 14.76	90.33 ± 13.93
FVC [% pred.]	106.10 ± 12.51	102.19 ± 15.32	108.62 ± 12.44	99.81 ± 14.23
FEV_1_/FVC	0.75 ± 0.07	0.74 ± 0.07	0.74 ± 0.07	0.74 ± 0.06
DLCOc [% pred.]	-	93.25 ± 16.33	-	94.15 ± 17.58
BAL-L%	32.6 ± 16.74	28. 88 ± 17.10	36.48 ± 16.38	24.99 ± 15.90
BALF CD4^+^/CD8^+^	7.60 ± 4.55	4.78 ± 4.12	8.39 ± 4.56	4.42 ± 3.68
Ca^2+^S [mmol/l]	2.43 ± 0.08	2.38 ± 0.23	2.39 ± 0.17	2.43 ± 0.15
Ca^2+^U [mmol/24 h]	5.15 ± 2.33	4.64 ± 2.96	4.27 ± 2.60	5.51 ± 2.67

*Abbreviations*: BALF, bronchoalveolar lavage fluid, *BAL-L%* % of BAL lymphocytes, *CD* cluster of differentiation, *DLCOc* lung diffusion capacity for carbon monoxide corrected for haemoglobin concentration, *FEV*_*1*_ forced expiratory volume in first second of expiration, *FVC* forced vital capacity, *pred* predicted, *S* serum, *U* urine

The control group for the study consisted of 23 healthy non-smokers (for concentration in BAL fluid) referred for bronchoscopy due to chronic cough or undefined changes on chest X-ray and 20 healthy non-smokers (for concentration genes in serum). The BAL control group consisted of subjects who, after thorough examination, were finally diagnosed either with idiopathic cough or as potentially healthy—when radiological signs were defined as clinically insignificant changes or artefacts.

All participants signed an individual consent form. The study was approved by the Ethics Committee of the Medical University of Lodz (RNN/141/10/KE).

### Bronchoscopy and bronchoalveolar lavage fluid (BALF) collection

Bronchoscopy was performed with a flexible bronchoscope (Pentax Safe-3000A, Tokyo, Japan) according to Polish Respiratory Society guidelines [19]. Patients optionally received Midanium and atropine before the examination; 2 % lidocaine was used as a topical anaesthetic. BAL fluid (BALF) was collected from the medial lobe, by instillation and subsequent withdrawal of 4 x 50 ml of 0.9 % NaCl. The fluid recovery was 52.1 ± 1.2 %. The crude BALF was filtered through a nylon gauze to clear the thick mucus and other contaminants, and then it was centrifuged at 2500 rpm for 10 min at room temperature. The supernatant was separated, frozen, and stored at −80 °C.

### Lung function tests

Spirometry was performed according to Polish Respiratory Society guidelines [20] with a computer-based spirometer (MES-1000, Poland). Forced vital capacity (FVC) and forced expiratory volume in 1 s (FEV_1_) were measured, and the Tiffenau index (FEV_1_/FVC) was calculated. Lung diffusion capacity for carbon monoxide was measured in patients presenting radiological signs of lung parenchymal disease only (stage II-IV) with a single breath method (Lungtest 1000 SB, MES, Poland) according to ATS/ERS standards [21]. The values were corrected for the haemoglobin concentration (DLCOc). All data (except the Tiffeneau index) were presented as the percentage of predicted value.

### Computed tomography

High-resolution, thin section CT (HRCT) scans of the lungs were available for 20 patients (out of 27 patients with stage II and III, 74 %). Sections of 1-mm collimation were acquired at 10-mm intervals from the apex to the dome of a diaphragm, at 120 kV, 200 mA, and a scan time of 0.6 s. All scans were obtained at full inspiration, and the images were obtained at a window level of −700 Hounsfield units and a window width of 900 Hounsfield units. For estimation of the extent of parenchymal involvement and hilar lymph node enlargement, the classification published by Drent et al. [22] was applied. Briefly, the extent of bronchovascular bundle thickening, parenchymal nodules, septal/non-septal lines, and parenchymal consolidation were estimated in a four-grade scale, and the sum of points described parenchymal involvement (total parenchymal score).

### Serum collection

The blood was collected from a cubital vein and left at 37 °C until clot formation (about 30–45 min). Then it was placed in a refrigerator at a temperature of 4 °C for several hours (0.5–24 h) until the total organisation of the clot. Next, the tube was centrifuged (1200 x g, 10 min, 4 °C), and serum was separated from the clot carefully into new sterile tubes, frozen, and stored at −20 °C.

### Immunoexpression analysis

TGF-β_1_, Smad 2, Smad 3, Smad 7, and VEGF-A immunoexpression levels in serum and BAL supernatant were assessed using the following: ELISA Kit for TGF-β1 ELISA Kit (650.010.096, Diaclone, Besancon Cedex, France), Enzyme-linked Immunosorbent Assay Kit For Mothers Against Decapentaplegic Homolog 2 (Smad 2) (SEC124Hu Cloud-Clone Corp. Huston, TX), Enzyme-linked Immunosorbent Assay Kit For Mothers Against Decapentaplegic Homolog 3 (Smad 3) (SEC123Hu Cloud-Clone Corp. Huston, TX), Enzyme-linked Immunosorbent Assay Kit For Mothers Against Decapentaplegic Homolog 7 (Smad 7) (SEA648Hu Cloud-Clone Corp. Huston, TX), and Enzyme-linked Immunosorbent Assay Kit For Vascular Endothelial Growth Factor A (VEGF-A)—(SEA143Hu Cloud-Clone Corp. Huston, TX). The intensity of the final colorimetric reaction in proportion to the amount of protein bound was measured on a plate reader (ELx800, BioTek) at 450 nm. The obtained results were calculated using a calibration curve constructed from standard solutions of known concentrations (0.001–10 ng/ml).

### Statistical analysis

Kruskal–Wallis test, Mann–Whitney test, Neuman–Keuls’ multiple comparison test, and Spearman’s rank correlation were used to assess the correlation between protein expression and classification of sarcoidosis based on radiological examination (stages I vs. II-III), acute vs. chronic disease onset, spirometric parameters, DLCOc, serum Ca^2+^ concentration, Ca^2+^ loss in 24-hour urine collection, the percentage of lymphocytes in BAL (BAL-L%), the phenotype of BAL immune cells (CD4^+^/CD8^+^), high-resolution computed tomography score, age, and sex of patients. *P* < 0.05 was established as the level of statistical significance. All calculations were performed by StatSoft (Poland).

## Results

### Immunoexpression levels of the studied proteins (TGF-β_1_, Smad 2, 3, 7, VEGF-A) in serum of sarcoidosis patients vs. controls

**Fig. 1:**
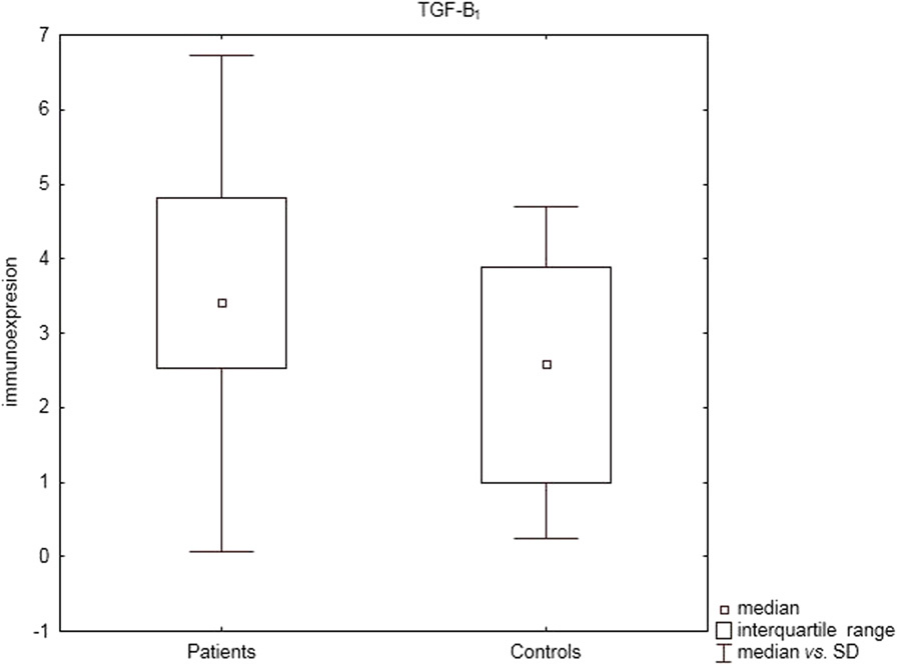
Box and whisker plots presenting statistically significant differences between patients and controls for TGF-β_1_ (*P* = 0.03, U Mann- Whitney test)

**Fig. 2:**
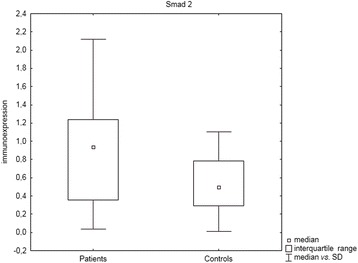
Box and whisker plots presenting statistically significant differences between patients and controls for Smad 2 (*P* = 0.01, U Mann–Whitney test)

**Fig. 3:**
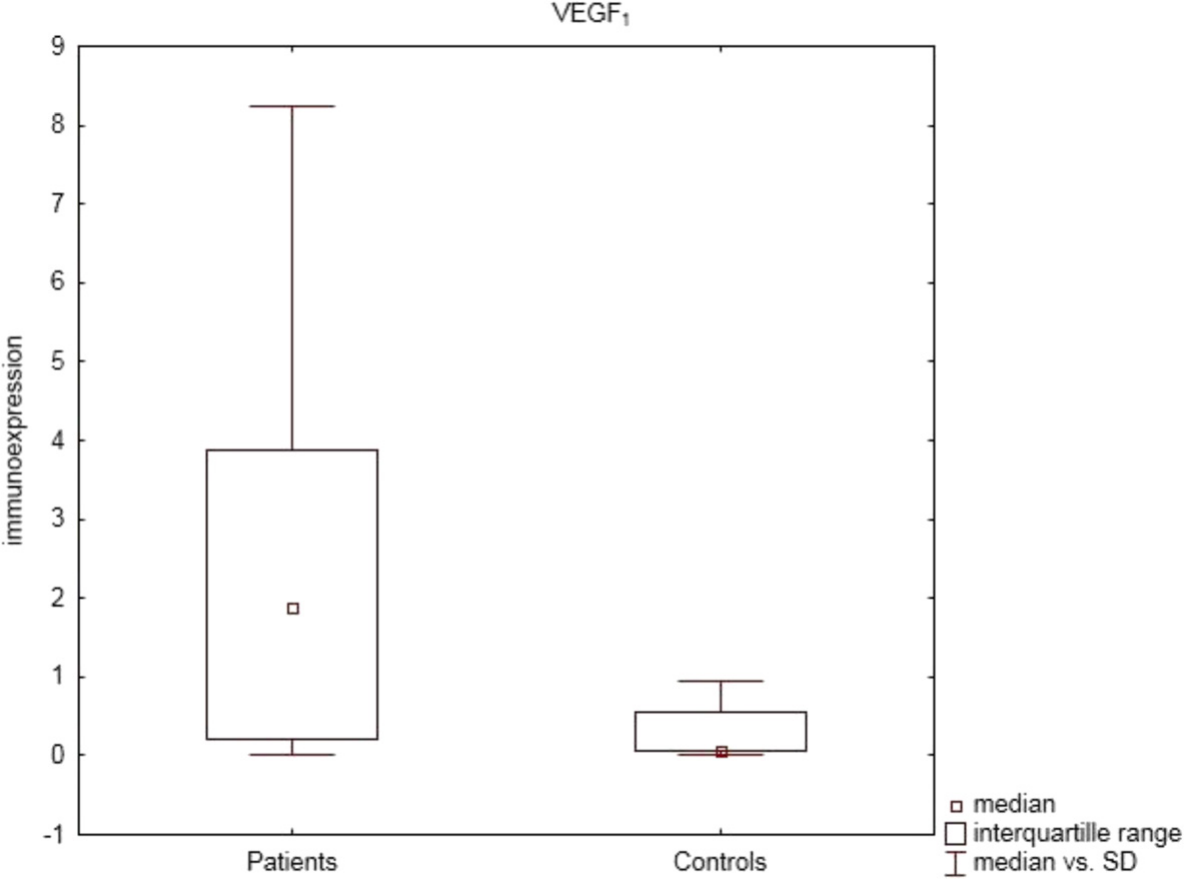
Box and whisker plots presenting statistically significant differences between patients and controls for VEGF-A (*P* = 0.0002, U Mann–Whitney test)

Statistically significant differences between patients and controls were observed for TGF-β_1_ (*P* = 0.03, U Mann- Whitney test), Smad 2 (*P* = 0.01, U Mann–Whitney test) and for VEGF-A (*P* = 0.0002, U Mann–Whitney test), with higher immunoexpression levels in sarcoidosis patients (see Figs. 1, 2, and 3).

### Immunoexpression levels of TGF-β_1_, Smad 2, 3, 7, and VEGF-A in serum and BAL fluid of patients with pulmonary sarcoidosis

In the serum of the studied patients the highest immunoexpression level was observed for TGF-β_1_ and lower for VEGF-A regardless of the presence of parenchymal involvement or clinical phenotype (acute onset vs. insidious onset). The lowest expression was found for Smad proteins, with significant lower mean immunoexpression value for Smad 7—see Table 2 for results.

**Table 2:** Immunoexpression (mean value, ng/ml) of all studied proteins (TGF-β_1_, Smad-2, −3, and −7, and VEGF-A) in serum and BAL supernatant of patients with sarcoidosis

Immunoexpression (mean value, ng/ml)	Serum	Protein	X-ray stage I	X-ray stages II-III	Acute onset	Insidious onset
		TGF-β_1_	3.58 (1.054–6.05)	3.40 (0.0625–6.728)	3.45 (0.0625–6.05)	3.53 (0.0625–6.728)
		Smad2	1.07 (0.244–4.784)	0.89 (0.035–3.71)	0.958 (0.035–4.784)	1.029 (0.061–3.71)
		Smad3	0.875 (0.061–9.187)	0.864 (0.061–6.846)	0.908 (0.061–9.186)	0.824 (0.061–6.846)
		Smad7	0.131 (0.058–0.681)	0.278 (0.058–2.82)	0.126 (0.19–0.681)	0.278 (0.058–2.827)
		VEGF-A	2.47 × 10^−3^ (0.55–8.25)	2.27 × 10^−3^ (0.55–10.28)	2.314 × 10^−3^ (0.55–8.25)	2.44 × 10^−3^ (0.55–10.28)
	BAL fluid	TGF-β_1_	0.064 (0.062–0.081)	0.075 (0.062–0.143)	0.06 (0.06–0.139)	0.07 (0.06–0.143)
		Smad2	not detected	not detected	not detected	not detected
		Smad3	not detected	not detected	not detected	not detected
		Smad7	not detected	not detected	not detected	not detected
		VEGF-A	0.0055 × 10^−3^ (0.0055–0.006)	0.01 × 10^−3^ (0.055–0.118)	0.0056 × 10^−3^ (0.0055–0.0072)	0.01 × 10^−3^ (0.055–0.118)

*Abbreviations*: *Smad*, (homologs of the Drosophila protein “mothers against decapentaplegic” (MAD) and the C. elegans protein SMA), *TGF* transforming growth factor, *VEGF* vascular endothelial growth factor

Also, in the BAL supernatant of the studied patients the highest mean immunoexpression level was observed for TGF-β_1_ and the lowest for VEGF-A (see Table 2). In BAL supernatant the concentration of Smad 2, 3, and 7 proteins were below the limit of detection (0.0054 ng/ml, 0.061 ng/ml, and 0.058 ng/ml, respectively).

There were no correlations (Spearman’s rank correlation test) between serum and BAL immunoexpression of TGF-β_1_ and VEGF-A (not calculated for Smads due to undetectable levels in BAL).

### Immunoexpression of TGF-β_1_, Smad 2, 3, and 7, and VEGF-A within sarcoidosis patients in relation to parenchymal involvement, clinical phenotype, and lung function test results

Statistically significant differences in immunoexpression levels of the studied proteins between patients without and with parenchymal involvement (stages I vs. II-III) and between clinical phenotypes (acute vs. insidious onset), both in serum and in BAL fluid, were not found (*P* > 0.05, U Mann–Whitney test).

Similarly, statistically significantly differences in immunoexpression levels of selected proteins in serum and BAL fluid were not found between patients with abnormal compared to normal spirometry or between patients with restrictive or obstructive spirometric pattern and normal spirometry (P > 0.05, U Mann–Whitney test).

### Immunoexpression of TGF-β_1_, Smad 2, 3, and 7, and VEGF-A within sarcoidosis patients in relation to patients’ gender and age

Higher expression of VEGF-A in the serum of men compared to women was found in the subgroup of patients with parenchymal involvement (*P* = 0.02, U Mann–Whitney test).

### The correlations between immunoexpression of the study proteins and selected biochemical and immunological markers and lung function parameters in serum and BAL fluid

We found few correlations between lung function parameters, selected laboratory markers, and protein levels in serum and BAL fluid of patients with pulmonary sarcoidosis (Table 3).

**Table 3:** Correlations between the immunoexpression levels of the studied proteins and lung function test parameters, and selected laboratory markers in patients with pulmonary sarcoidosis

Biological material	Parameter	Protein	Subgroup	Rho value	*P* value
Serum	Ca^2+^U	TGF-β_1_	Acute onset	0.494	0.0437
	FEV_1_/FVC	VEGF	Parenchymal involvement	−0.488	0.0113
BAL fluid	FVC	TGF-β_1_	Abnormal Spirometry	−0.702	0.0234

*Abbreviations*: see Tables 1 and 2 legends

### The correlation between protein immunoexpression levels and high-resolution computed tomography score

Spearman’s rank correlation coefficient revealed statistically significant positive correlations between Smad 2 level in serum and HRCT scores: HRCT score (parenchymal), HRCT (nodular abnormalities), HRCT (linear abnormalities), HRCT (consolidation) (see Table 4).

**Table 4:** Correlations between the immunoexpression levels of Smad 2 and high resolution computed tomography (HRCT) in patients with pulmonary sarcoidosis

Biological material	Parameter	Protein	Rho value	*P* value
Serum	HRCT score (parenchymal)	Smad2	0.533	0.0187
	HRCT(nodular abnormalities)	Smad2	0.476	0.0389
	HRCT (linear abnormalities)	Smad2	0.508	0.0263
	HRCT (consolidation)	Smad2	0.491	0.0324

## Discussion

To sum up, we found that patients with sarcoidosis have higher serum levels of TGF-β1, Smad 2, and VEGF-A than control subjects. Such a difference was not found in BAL fluid, which is striking taking into account the known phenomenon of peripheral depletion of CD4 lymphocytes related to accelerated inflammatory reaction at the site of granuloma formation, i.e. in the lung. T regulatory cells (CD4 + CD25 + FOXP3+ and CD4 + CD39+) may be involved because they incompletely control the inflammation of injured tissues but are powerful enough to mediate peripheral anergy [23, 24]. In this context, our findings speak against this commonly acknowledged concept. It is possible, however, that peripheral anergy is related merely to the interplay between various subsets of lymphocytes, but different inflammatory mediators are released from different cellular sources (both tissue-settled and circulating inflammatory cells) into the circulation. This is in line with the concept of sarcoidosis as a systemic disease. The lack of correlations between these proteins in peripheral blood and BAL fluid seems to confirm that these processes are far more complicated, and that the peripheral concentration of these markers does not directly reflect their local production at the site of inflammation. Similar conclusions were also drawn by other authors, who found that peripheral blood mononuclear cells (PBMNC) isolated from sarcoidosis patients released higher amounts of various cytokines involved in the pathogenesis of sarcoidosis (such as IL-1β, TNF-α, IL-6, and GM-CSF), compared to controls [25].

Also, locally the net production of TGF-β is not a result of the activity of one-cell type. It was shown, that BAL fluid TGF-β concentration is not related to its production by isolated and cultured alveolar macrophages (AM) in vitro [11], and in vitro production of this cytokine by both peripheral and BAL mononuclear cells is not related to any in vivo radiological and functional disease parameter [25].

In our study we observed the highest detected levels in both serum and BAL fluid for TGF-β, and the lowest for VEGF-A. There were no differences between subgroups of patients classified according to the presence or absence of parenchymal disease, or lung function tests results. Only the negative correlations between BAL TGF-β and FVC (only in patients with abnormal spirometry) support previous data confirming the negative prognostic value of this biomarker [11]. The lack of clear-cut differences between patients with and without the presence of acknowledged risk factors connected to worse prognosis, such as parenchymal involvement and functional impairment, may be a result of selection bias. Our group of patients were randomly selected (all consecutive patients with confirmed diagnosis of sarcoidosis diagnosed in our department) and consisted mainly of patients with relatively good prognosis. More than 50 % of patients did not show radiological signs of parenchymal involvement, there were no patients with stage IV disease, and no patients had severe lung dysfunction. Although a positive correlation was found between urine calcium loss and serum immunoexpression level of TGF-β_1_, only few patients revealed impaired calcium metabolism. Moreover, this is a “one time point” study, and follow-up data on the prognosis are not available at this stage.

TGF-β pathway may be involved in self-remitting disease, when early phase of inflammation, characterised among others by accelerated TNF-α, IFN-γ, and IL-2 production, gradually expires and its activation may herald disease remission. On the other hand, the TGF-β pathway may be activated in progressive lung fibrosis. Indeed, TGF-β/Smad signalling has been recognised as a key mediator of fibrosis. Therefore, in our study we also focused on Smad protein immunoexpression levels in sarcoidosis patients. We confirmed that Smad 2 immunoexpression was significantly increased in serum (but not in BAL fluid) of sarcoidosis patients as compared with controls. It should be pointed out that the data for Smad proteins in sarcoidosis are lacking, but it may be presumed that the trends for Smad (Smad 2 and/or 3) immunoexpression should follow that of TGF-β_1_. It has been well documented that Smad 2 and 3 are key downstream mediators of TGF-β_1_ and their biological function is the regulation of fibrogenesis in response to TGF-β_1_. However, Smad 2 and Smad 3 activation and/or TGF-β responses may be altered in different human cell types because of differences in binding of Smad 2 and 3 to adaptor proteins SARA (Smad anchor for receptor activation). This mechanism may be responsible for the divergence between Smad 2 and Smad 3 immunoexpression and activation. Furthermore, the differential activation of Smad 2 and Smad 3 can be connected with a unique subset of protein interactions with the TGF-β receptor complex [26]. Indeed, in our study we did not find mutual correlation between the immunoexpression of these proteins, but we observed that serum Smad 2 immunoexpression was elevated in sarcoidosis patients. Interestingly, in our study we observed a positive correlation between HRCT scores and overexpression of Smad 2. Recently, similarly to our data, intensive immunoexpression of Smad 2/3 complex has been observed in an animal model in dogs with IPF [27].

Taking into account that VEGF is a pleiotropic cytokine and strong proangiogenic mediator, and additionally that it is observed in the areas of granuloma formation [18, 28], in our study we decided to assess the immunoexpression level in sarcoidosis patients. Granulomas are prominent mainly in the early phase of the disease, whereas in chronic disease they are replaced by fibrosis. VEGF-A has also been shown to be involved in lung fibrosis. We confirmed that VEGF-A was increased in serum (but not in BAL fluid) in patients, regardless of clinical and radiological classification. Our results are in contrast to those obtained by Porębska et al. [29], who did not find differences in the level of immunoexpression between patients and healthy subjects in serum. Insignificant differences were obtained only in BALF [29]. Our results are inconclusive in this matter, showing increased immunoexpression of VEGF-A in serum, but we were not able to show any correlations with parenchymal involvement and lung function test impairment. Zielonka et al. [30] found negative correlation between serum angiogenic activity of ILD patients and their lung diffusion capacity. A comparison of VEGF-A concentration in BAL fluid retrieved from lung segments with more and fewer intensive parenchymal changes revealed higher concentrations of VEGF-A in more involved lung areas [31]. In sarcoidosis, data on the involvement of angiogenesis and angiostasis in different stages of disease natural history are sometimes contradictory. The angiostatic microenvironment was described in severe sarcoidosis patients by several authors [15].

## Conclusions

It should be stressed that the up-regulation of VEGF-A and TGF-β_1_ in sarcoidosis patients observed in our study, as compared with healthy subjects, supports data confirming the involvement of these proteins in the pathogenesis of sarcoidosis. However, their usefulness as markers in the clinical aspect is controversial and requires further study, particularly when TGF-β seems to be modulated via many specific cytoplasmic or membrane-bound proteins (including Smad 2 and/or Smad 3), which are under regulation of different post-transcriptional and translational modifications. Our observations concerning increased Smad 2 immunoexpression level in patients, and its correlation with HRCT indicates the potential significance of Smad 2—but not Smad 3—in the clinical course of sarcoidosis. On the other hand, the lack of mutual correlation between Smad 2 and Smad 3 supports the thesis that Smad 2 and Smad 3 may separately regulate different tissue-specific activation of TGF-β_1_ via various mechanisms, which determine fibrosis and immune response in the lungs.

## Abbreviations

BAL: Bronchoalveolar lavage
BALF: BAL fluid
DLCO: Diffusion capacity for carbon monoxide
EBUS-TBNA: Endobronchial ultrasonography guided transbronchial needle aspiration
ELISA: Enzyme-linked immunosorbent assay
FEV_1_: Forced expiratory volume in first second of expiration
FVC: Forced vital capacity
HIF: Hypoxia-inducible factor
ING: Inhibitor of growth factor
LFT: Lung function test
TGF: Transforming growth factor
VEGF: Vascular endothelial growth factor
